# A systematic review of the definitions, narratives and paths forwards for a protein transition in high-income countries

**DOI:** 10.1038/s43016-023-00906-7

**Published:** 2024-01-03

**Authors:** Océane Duluins, Philippe Vincent Baret

**Affiliations:** SYTRA, Earth and Life Institute—Agronomy, Université catholique de Louvain (UCLouvain), Louvain-la-Neuve, Belgium

**Keywords:** Environmental studies, Sustainability

## Abstract

The protein transition, aiming to rebalance protein intake between animal and alternative proteins, is gaining momentum in scientific and policy discussions on food system transformation. Here, using a systematic review approach, we identified 33 articles that address challenges in reducing the environmental impacts of protein production and consumption, providing healthy diets for a growing population and preventing adverse effects of industrial livestock production systems. We found unclear definitions of the protein transition, conflicting views on reduction or replacement of dietary protein and a lack of attention to systemic change by reducing protein to its macronutrient function. Three narratives were identified, namely, the consumer narrative focusing on consumption-based solutions targeting dietary changes; the techno-centred narrative developing new, more resource-efficient protein production systems; and the socio-technological narrative that intends to transition the agri-food system from an animal-dominated regime to an alternative protein regime. We conclude that solutions should consider factors such as scale, initiating actors and expected impact to support complementary protein transition approaches.

## Abstract (web summary)

Protein production and consumption changes are needed to support healthy and sustainable food transformations. This systematic review explores narratives, gaps in the literature and paths forwards by focusing on the protein shift as a transition process in high-income countries.

## Main

Proteins play a central role in diets and are crucial in ensuring nutrition. They come from various sources, including plant and animal products^1^. While animal protein consumption tends to increase with economic development in low- and middle-income countries, its role is increasingly contested in high-income countries^2,3^. During the past decade, the scientific community has highlighted the negative externalities associated with the overproduction and overconsumption of animal proteins, including impacts on the environment, human health and animal welfare^4–6^. In this regard, there is a growing discussion on the impacts of the prevailing ways of producing proteins on the long-term sustainability of food systems. This eventually resulted in conceptualizing a ‘protein transition’ and integrating the concept into scientific and societal debates.

The protein transition is approached from various perspectives in scientific research. One strand of the literature explores the future of proteins in general, encompassing all protein sources. This strand of research includes discussions about alternative protein sources, debates around livestock and its substitutes, and varying visions for the future of protein consumption^7,8^. Another strand of research focuses specifically on alternative proteins, including their production, consumption, acceptance, environmental impact and associated narratives^9–12^. Finally, numerous studies explore the future of animal proteins and their role in food systems, investigating the effects of achieving multiple targets on animal protein production and consumption^13,14^. A previous study^8^ analysed sustainability issues related to all protein sources. However, this study did not use a fully systematic approach to article selection because of the scattered literature on proteins and the overwhelming number of sources.

Our contribution is twofold. First, while most papers focus on specific protein sources as an entry point, we choose a more global approach by focusing on the protein shift as a transition process. Second, our systematic review comprehensively analyses all articles published on the protein transition, ensuring a balanced presentation of the diverse narratives and their salient features.

The protein transition is a hot topic discussed at different levels of societal organization, including political discussions, private company strategies and public coverage in the media^8,15,16^. At the political level, several initiatives and strategies promote the protein transition as part of a transition towards more sustainable food systems. These include the Farm to Fork Strategy (European Green Deal), the Canadian food policy, the Brazilian National Plan for Agroecology and Organic Production, the Healthy China 2030 Plan and the United Nations Sustainable Development Goals. At the private sector level, the protein transition has attracted increased private investment, speculative finance (including high-risk trading), innovation and product development^17–19^.

The fact that the protein transition is an unstable emerging theme used by different actors and for various purposes calls for a clarification of the concept and the underlying scientific evidence, as well as the identification of both knowledge gaps and points of contention and debate.

The objective of this systematic review is threefold:

1. Identify definitions and interpretations of the protein transition concept (see ‘The protein transition definitions’ in Results)
2. Identify the key challenges that the protein transition promises to address (see ‘The three challenges protein transition aims to address’ in Results)
3. Identify the narratives comprising diverse solutions to achieve the protein transition (see ‘The emergence of three narratives’ in Results)

This Article focuses on Organisation for Economic Co-operation and Development (OECD) countries as they are typically high-income nations with relatively high levels of meat consumption^20^. Therefore, studying these countries can provide insights into potential environmental and health benefits of reducing meat consumption^6^. In addition, their global political and economic influence may have ripple effects on the global food system. While relevant in high-income settings, the protein transition concept is unsuitable for regions where diets are nutritionally inadequate and characterized by a lack of animal protein intake^21^.

Throughout the Article, we will refer to alternative proteins as protein sources including insects, algae, plant-based single-cell proteins and fungi, as opposed to ‘traditional’ animal proteins such as meat, fish, dairy and eggs.

The review reveals shortcomings that could hinder the protein transition, including a poorly defined concept and the overlook of complexity. In the discussion, we highlight the need to establish links between the proposed solutions and specific challenges. In addition, we stress the importance of understanding the extent to which these solutions contribute to the targets of the protein transition.

## Results

### The protein transition definitions

**Fig. 1:**
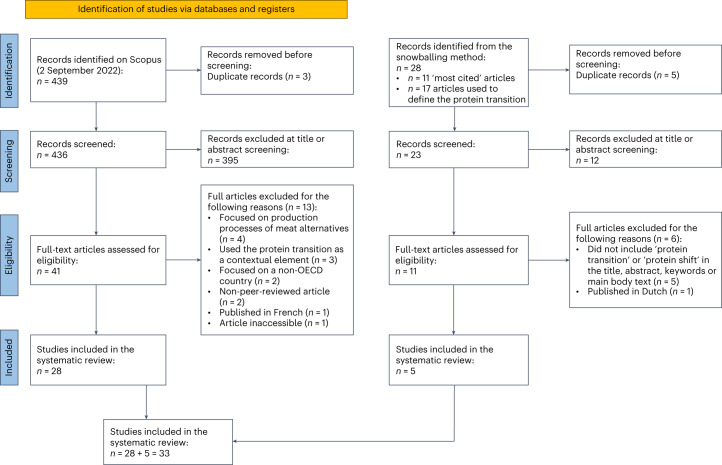
PRISMA workflow. PRISMA workflow diagram: the left side shows database-identified articles, and the right side presents records from the snowballing method. Four review phases are outlined for each method, along with corresponding article numbers. Figure adapted with permission from ref. ^66^ under a Creative Commons license CC BY 4.0.

We identified 33 studies for inclusion (Fig. 1) based on the eligibility criteria (Methods). Out of the 33 articles reviewed, 17 included an explicit definition of the protein transition, 3 provided an implicit definition and 13 did not define the protein transition (Supplementary Table 1).

Out of the 20 (17 + 3) articles providing a definition, 16 defined the protein transition from a consumption perspective, agreeing on the following definition: a shift from a diet rich in animal proteins to one richer in alternative protein intakes. Only one article mentioned a reduced total protein intake^22^. Of the 20 articles providing a definition, 15 mentioned plant proteins, while 5 referred to alternative proteins, generally including plant proteins.

By design, the protein transition affects protein production and consumption^23^. However, only two articles explicitly included a production dimension in their definitions, stating that the transition should allow a reduction in consumption and production^24,25^.

### The three challenges protein transition aims to address

**Fig. 2:**
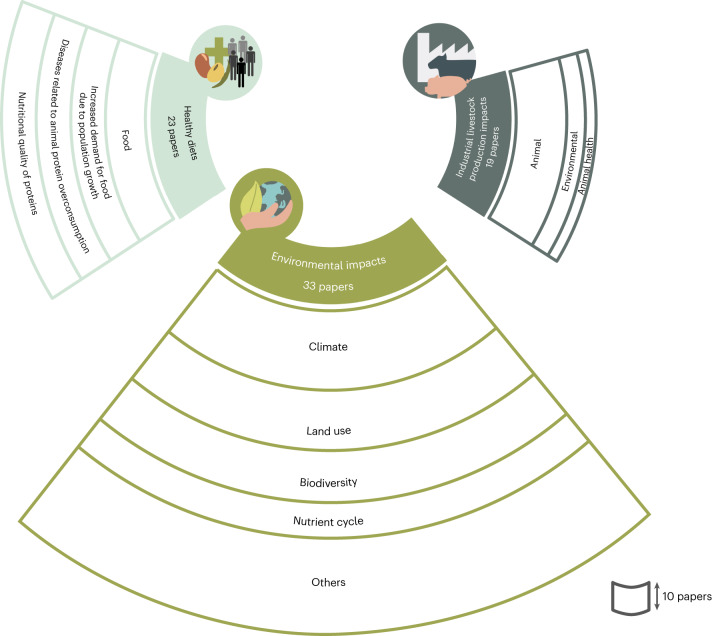
Challenges referenced in selected articles and identified via abductive thematic analysis. The frequency of challenges mentioned across articles, with coded sub-themes for each challenge; the thickness of the strips reflects the frequency of sub-theme occurrences.

Three main challenges the protein transition aims to address were identified (Fig. 2). These challenges provide the rationale for the necessity of the protein transition and are not mutually exclusive.

#### Reducing the environmental impacts of protein production and consumption

All articles mentioned the environmental impacts of protein production and consumption, particularly animal protein. Environmental impacts include greenhouse gas emissions and their contribution to climate change, biodiversity loss, and nitrogen and carbon cycle disruption^26–29^. This challenge emphasizes that protein production and consumption patterns are outside the safe operating space defined by the planetary boundaries^30,31^.

#### Providing healthy diets for a growing population

Although focusing on high-income countries, nearly half of the articles cited the need for a protein transition due to population growth and food security. The global population and the resulting demand for animal proteins are projected to increase in the coming years, with some regions already overconsuming animal proteins^27,28,30–37^. This increase in demand will increase the pressure on food security (that is, the moment “when all people, at all times, have access to sufficient, safe, and nutritious food to meet their dietary needs for an active and healthy life”)^37^. The challenge is presented in the articles as being twofold: (1) producing enough food and providing adequate diets for the entire population in a finite-resource world and (2) addressing the negative health impacts of overconsuming proteins (particularly red and highly processed meats, which increase the risk of diseases such as type 2 diabetes, cardiovascular disease and certain types of cancer).

#### Preventing the ethical problem of animal welfare in industrial livestock production systems

About a third of the articles view the protein transition as a critical step towards reducing the negative externalities associated with industrial livestock production systems (ILPS). Such systems are generally associated with poor animal welfare owing to overcrowding and restraining animals indoors for their entire lives, which raises ethical concerns^38,39^. ILPS are linked to intensive antibiotic use, leading to antibiotic resistance and increasing the incidence of emerging diseases affecting animal and human health (for example, avian influenza or bovine spongiform encephalopathy)^26,27^. ILPS are presented as being associated with the most important adverse environmental impacts^28,32,37–39^. They typically rely on high-quality feed protein, such as maize and soy feed, leading to further negative environmental effects like deforestation and high pesticide use^36,39^.

### The emergence of three narratives

Based on solutions proposed in the articles (Supplementary Table 2), we identified three narratives associated with the protein transition. Narratives create a coherent discourse from the world-views and beliefs^8,40^. They are the primary means of conveying importance and meaning and play an important role in mediating between the individual and society. In this Article, a narrative can be described by combining a driver of change (that is, the main issue to be addressed), the objective regarding the desirable future and one or more action pathways (Table 1). The most frequent narratives are the consumer narrative (*n* = 13), followed by the techno-centred narrative (*n* = 10), then the socio-technological transition narrative (*n* = 8). Two articles remain unclassified as the solutions proposed in those articles did not fit one of the narratives. In one of the articles^41^, a gap between the research focus and proposed solutions was observed, which did not align with any identified narratives. On the other hand, another study^42^ presented solutions that intersect between two narratives, making it challenging to categorize them distinctly.

**Table 1:** The three main narratives identified include the driver of change, the main objective pursued and the action pathways

Narrative	Driver of change	Main objective	Scale of intervention	Initiating actors	Action pathways
Consumer narrative	Unsustainable consumption patterns	Dietary shifts	Micro, defined as the consumer level	Consumers, civil society	• Reducing and substituting animal proteins • Changing to alternative diets
Techno-centred narrative	Inefficient protein production systems	New, more resource-efficient novel protein production systems	Meso, defined as the value chain level	Value chain actors, including industry	• Research and development • Infrastructure and technology
Socio-technological transition narrative	Unsustainable food protein ‘regime’	Agri-food system transition	Macro, defined as the regime level	Research, civil society, governments, private sector actors	• Redefining the food system regime • Redirecting public and private financial support • Implementing new regulatory frameworks

In the techno-centred narrative, inefficiency is the driver of change. It focuses on enhancing the efficiency of resources needed per unit of protein (for example, land and water). The socio-technological transition narrative is motivated by the need to address the unsustainable protein regime, as defined by previous studies^63–65^. The regime denotes the prevailing and stable socio-technical system shaped by cultural norms, world-views and embedded structures, including physical infrastructure, laws, regulations and policies.

The three narratives do not address the same challenges or imply the same policy priorities (Table 2).

**Table 2:** The main challenges highlighted and policy priorities across various narratives

Narrative	What challenge or challenges are emphasized?	Main policy priorities associated with the narrative
Consumer narrative	• Providing healthy diets for a growing population • Reducing the environmental impacts of protein production and consumption	• Taxes and subsidies to incentivize changes in consumption patterns • Labelling and certification to help consumers make informed choices • Education and awareness on the benefits of reducing animal protein consumption
Techno-centred narrative	• Reducing the environmental impacts of protein production and consumption • Preventing the ethical problem of animal welfare in industrial livestock production	• Research and development in alternative proteins • Funding and subsidies towards alternative proteins • Adapted regulatory frameworks for novel protein sources • Public–private partnerships
Socio-technological transition narrative	• Reducing the environmental impacts of protein production and consumption • Providing healthy diets for a growing population • Preventing the ethical problem of animal welfare in ILPS	• Holistic policy framework overcoming political silos • Regional and national coordinated action plans involving multi-stakeholder collaboration (for example, governments, civil society organizations and private sector actors)

### The consumer narrative

**Fig. 3:**
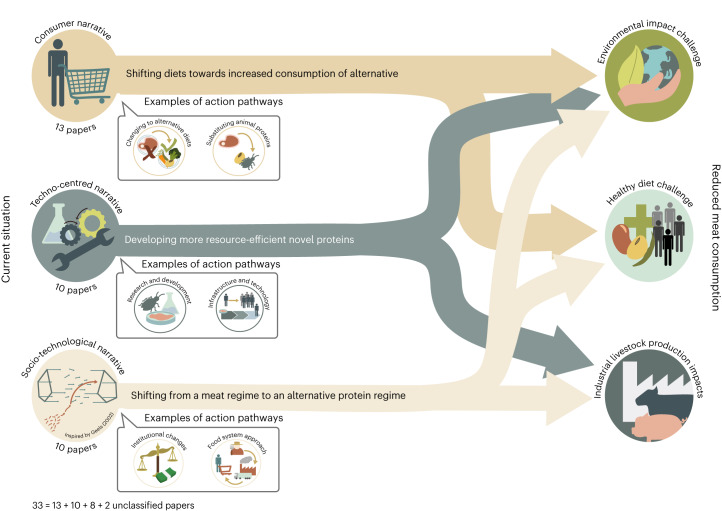
The three narratives of the protein transition. The left side depicts the present state and narratives of the protein transition, featuring examples of action pathways. The right side shows the primary challenges targeted by the protein transition. The lines represent the interactions between narratives and challenges.

This first narrative is centred on consumer-focused solutions (Fig. 3). Its main goal is to shift dietary habits from animal proteins to alternative protein sources^32^. The solutions proposed in this narrative are specifically geared towards a micro-scale, defined at the consumer level, with consumers and civil society emerging as the primary initiators of this narrative (Table 1). Strategies to reduce the consumption of animal proteins and encourage the adoption of alternatives include information campaigns to raise awareness and educate consumers^15,32,43^; coercion and incentives such as fiscal policies or price signals^26,27,44,45^; promoting smaller portions of meat, meatless days and consuming better-quality meat in smaller quantities^35,44,46^; nudging^28^; training and improving cooking skills^44^; persuasive campaigns highlighting the benefits of alternative dietary habits^44,45^; and inspiring individuals through modelling^45^ (Table 3). Some articles also focus on consumers adopting alternative dietary habits, such as flexitarianism or vegetarianism^33–35,47^, while others focus on the acceptability of dietary shifts and substitution strategies implemented in practice^48^ (Table 3). The consumer narrative, therefore, emphasizes behavioural and cultural approaches.

**Table 3:** Proposed solutions within each action pathway

Narrative	Action pathways	Solutions
Consumer narrative	• Reducing and substituting animal proteins	• Information strategies • Coercion and incentivization • Promoting smaller portions of meat and meatless days • Eating less but better meat
	• Changing to alternative diets	• Promoting and switching to flexitarianism, vegetarianism and veganism • Training and improving cooking skills
Techno-centred narrative	• Research and development	• Cultivating, developing and commercializing alternative proteins for food and feed
	• Infrastructure and technology	• Developing value chains for alternative proteins • Investing in sustainable technology innovation
Socio-technological transition narrative	• Redefining the food system regime	• Redefining the role of and power relationships between breeders, lobbying, non-governmental organizations and retailers in both the current and desirable regimes • Scenario building of the transition
	• Redirecting public and private financial support	• Changing financial support for supply (for example, subsidies)
	• Implementing new regulatory frameworks	• Introducing new norms, rules, regulations and standards (for example, labelling regulations) • Changing public guidance on food consumption (for example, through dietary guidelines)

### The technocentric narrative

The second narrative focuses on solutions aimed at developing alternative proteins for food and feed through research, development, technology and infrastructure (Fig. 3). The scale of action of the technocentric narrative is situated at the meso level, defined as the value chain level, with the primary initiators being value chain actors, including industry stakeholders (Table 1). This narrative proposes alternative proteins for both human consumption and animal feed to make protein production more resource efficient and reduce negative impacts associated with current protein production. Solutions such as insects and seaweeds, which can efficiently convert low-quality by-products into biomass, are seen as potential replacements for soy feed imports and human food options^30,31,49^ (Table 3). Moreover, plant-based alternatives are viewed as more sustainable food options for humans^15,22,26,28,29,38,50^ (Table 3). Therefore, research and development efforts are encouraged to develop alternative options to animal-based foods^26,37,43,51,52^. In addition, technological innovations and infrastructure are seen as crucial means to advance the protein transition^52^, although they are unlikely to be sufficient to achieve the transition by themselves^24,35,53^ (Table 3).

### The socio-technological narrative

The last narrative is anchored in a perspective of reconfiguring the whole-food protein regime, currently dominated by the animal protein sector (Fig. 3). In this narrative, change must come not only from consumers but also from actors of the entire food system (for example, lobbies, retailers, governments)^43,50,54^. This narrative only mentions the need for a change in animal production (that is, potential shifts towards more sustainable livestock systems or livestock number reduction)^39,45^. This narrative delves into the role of trust and system networks in transition dynamics. For example, one study^25^ focuses on how the deliberate establishment of trust and a shared vision contribute to the protein transition, while another study^55^ examines alliance formation, its impact on system building and its potential to accelerate the transition. This narrative includes redefining the protein regime by envisioning changes required in the current protein regime (for example, using a backcasting approach)^43^. Implementing new regulatory frameworks plays a crucial role in fostering the transition, and solutions include introducing new norms, rules, regulations and standards^15,22^. Redirecting public and private financial support is equally pivotal in propelling this paradigm shift, affording the necessary impetus for transformative progress in sustainable food systems^39,43,45^. The socio-technological narrative targets actions towards a holistic policy framework and regional and national coordinated action plans involving multi-stakeholder collaborations^15,25,43^.

## Discussion

### Defining the protein transition

The protein transition is an unstable and emergent theme. The definition plays a crucial role in establishing the target, paving the way for various solutions to achieve this objective. In our systematic review of articles on the protein transition, we observed that 13 out of 33 papers failed to define this concept. For these articles, the protein transition was seen as an ongoing process assumed to be understood without needing specific clarification.

Articles defining the protein transition showcase diverse perspectives on this evolving theme. Some view it as a shift towards a broad range of alternative proteins (5 articles), while others restrict it to a shift towards plant-based proteins (15 articles). Views on the protein transition also differ regarding its association with reducing total protein intake or substituting animal proteins with alternative sources while maintaining current intake.

In all but two papers, food is reduced to a single macronutrient, hiding the diversity of origins of proteins. This reductionist approach can lead to misconceptions about the nutritional interchangeability of proteins and disregard the complexities of the human digestive system and metabolism^42,56,57^. Moreover, alternative proteins become another protein delivery system: their sole purpose is to replace animal proteins by providing proteins as similar as possible to animal ones^17^. This functional reduction overlooks the multiple roles husbandry provides, including the role of animals in the circular flow of materials in agriculture, ecosystem services and soil fertility management^58–60^. Lastly, the protein transition will entail a systemic change inconsistent with the functional reduction of different types of food to their role as micronutrients (for example, a change in diet or protein production and supply systems).

A poorly defined or undefined protein transition can be used to justify specific actions or solutions without adequately addressing the core challenges mentioned in ‘The three challenges protein transition aims to address’ in Results^18^. A clear definition is essential to ensure that the objectives of the protein transition are aligned with the identified challenges and that appropriate actions are taken to address them effectively.

### Towards a consensual definition

While finding a universally consensual definition of the protein transition might be a complex endeavour, it is important to consider that a well-crafted definition can facilitate and enhance engagement from diverse stakeholders, including territorial actors, public authorities and the private sector. The definition could benefit from clarifying the specific types of protein under consideration and delineating whether the emphasis lies in substitution or reduction. In addition, opting for a systemic approach would help avoid reducing the diverse food sources to merely their functional role as macronutrients.

### Navigating protein transition solutions

Each narrative plans to contribute to the protein transition, hoping to address the key challenges that have led to the need for this transition.

Two questions arise. First, do the proposed solutions address the challenges, and if so, how? Second, to what extent do these solutions contribute to meeting the specified targets?

Taking the first narrative as an illustrative case, this involves delving into how dietary adjustments directly address the challenges pinpointed in this systematic review. For instance, in what ways do alternative diets provide healthier and more environmentally friendly options? Furthermore, this inquiry entails quantifying the impact: what level of reduction in greenhouse gas emissions can be attained? Who stands to gain the most from these healthier dietary choices, and to what degree?

Firstly, the protein transition demands establishing clear links between proposed solutions and specific challenges. This alignment is frequently missing, making it impossible to ascertain whether the proposed solutions effectively address the identified challenges in the introduction sections of the papers included in the review.

Secondly, it is important to understand to what degree these solutions contribute to the targets of the protein transition. This review has highlighted a mismatch in the scale of issues outlined in the introductory contextual elements of papers, encompassing projections of a burgeoning global population of nine billion, heightened protein demand, the environmental repercussions of animal protein production and, on the other hand, the pragmatic, actionable solutions that often pertain to localized behavioural, dietary changes within specific countries and population groups.

Solutions would benefit from being located within the array of available options. This involves understanding where these solutions fit in terms of scale, the initiating actors and the anticipated impact. This discernment is crucial in forestalling the ex post justification of actions and fostering complementarity between different approaches and solutions.

## Concluding remarks

Three main narratives associated with the protein transition are identified in this systematic review. Each narrative exhibits different drivers of change, solutions and policy priorities for the future. The consumer narrative focuses on consumption-based solutions targeting dietary changes. The techno-centred narrative aims to develop new, more resource-efficient protein production systems. The socio-technological narrative intends to transition the agri-food system from an animal-dominated regime to an alternative protein regime. Policy priorities range from taxes and subsidies to incentivize changes in consumption patterns for the consumer narrative, to funding and subsidies for developing alternative proteins for the techno-centred narrative, to comprehensive policy frameworks to overcome political silos and promote a more integrated approach for the last narrative.

Advocated to address issues stemming from excessive animal protein consumption and production in high-income settings, the protein transition is presented as a sustainable option for the environment, human health, food security and animal welfare. It is crucial to establish a solid link between the protein transition and its impact on addressing core challenges and quantifying to what extent the solutions contribute to the targets. While increasingly used in the scientific literature, the protein transition concept lacks a consistent definition. This lack of clarity can pose potential issues as various perspectives on the protein transition emerge, reinforced by the diversity of actions and political priorities associated with the different narratives.

## Methods

Our main objectives centred on unravelling the various definitions, interpretations and narratives related to the protein transition. To achieve this, we focused on scientific literature, encompassing all articles pertaining to the protein transition. The literature review was conducted and reported according to the Preferred Reporting Items for Systematic Reviews and Meta-Analyses (PRISMA) guidelines to ensure a rigorous and transparent approach to our literature selection and analysis^61^. The flow chart summarizing the study selection is depicted in Fig. 1. We did not submit a review protocol to PROSPERO (or similar online databases) as this review does not address a health-related outcome.

### Identification and screening

The literature was searched in September 2022 on Scopus using the following terms: ‘protein transition’ OR ‘protein shift’ OR ‘sustainable protein consumption’ OR ‘sustainable protein production’. The fields searched included the title, abstract and keywords.

After screening titles and abstracts, we excluded articles not dealing with the protein transition, most of which focused on the physical properties of proteins (Fig. 1). Using the guidelines for snowballing in a systematic literature review^62^, we identified 28 additional articles (Fig. 1). The snowball method was performed by screening the most cited references within the articles’ introductions already included in the systematic review (Supplementary Table 3). All articles with more than five citations were screened for eligibility (Table 4).

**Table 4:** Inclusion and exclusion criteria for the selection of articles

Criteria	Inclusion	Exclusion
Keyword presence	Includes ‘protein transition’ or ‘protein shift’ in the title, abstract, keywords or main body text	Does not include ‘protein transition’ or ‘protein shift’ in the title, abstract, keywords or main body text
Peer review	Peer-reviewed article	Non-peer-reviewed article
Language	Published in English	Published in another language than English
Availability	Full text available online	Full text not available online (for example, books)
Definition	Focuses on a protein transition leading to a lower animal protein consumption (definition in high-income settings with a high protein intake)	Focuses on a protein transition towards increased consumption of animal proteins (definition in low-income settings with low protein intake) or unclear definition
Topic	Explicitly investigates at least one of the following topics: protein consumption, livestock production, behavioural change in diets and alternative proteins to meat	Focuses on the production processes of meat alternatives or alternative proteins, and the thermal properties of proteins, or takes the protein transition as an example or a contextual element
Geographical focus	Focuses on an OECD country	Focuses on a non-OECD country

All inclusion criteria had to be met for an article to be included, whereas only one exclusion criterion was required for an article to be excluded.

### Testing for eligibility and final inclusion

A total of 52 articles were thoroughly analysed and tested for eligibility using inclusion and exclusion criteria (Table 4). In total, 33 (28 + 5) articles were included after this step.

### Data coding and synthesis of the results

The analysis was conducted using NVivo 20.1 software, following an iterative coding process based on previous knowledge of the literature, which allowed us to test the first set of codes. We independently tested the initial coding proposal on five articles and subsequently refined the codes through comparison and discussion. Once the final codes were established, they were applied to all 33 papers. Additional information on the coding process can be found in ‘Coding Instructions’ in Supplementary Information.

The coded text was then organized into thematic tables (Supplementary Fig. 1), including protein transition definitions and interpretations (Supplementary Table 1), proposed solutions (Supplementary Table 2), citations in the introduction (Supplementary Table 3), article bibliometrics (Supplementary Table 4), research focus and methodology (Supplementary Table 5), problem framing (Supplementary Table 6) and sustainability themes (Supplementary Table 7). This thematic grouping allowed us to conduct further analysis to achieve our three research objectives: (1) identifying definitions, (2) understanding challenges and (3) exploring narratives related to the protein transition.

### Critical analysis of review design and potential biases

We conducted independent coding and analysis to minimize internal biases within the research team that could affect the exploratory and qualitative approach. In addition, we adopted a deductive–inductive approach to prevent restricting the results based on a preconceived coding framework. Main points of contention and debates arose when discussing the codes and the inclusion of specific articles.

It is worth noting that the search was restricted to material in the English language. This limitation is justified as English is the primary language of scientific publication in regions where the protein transition concept is most relevant. Finally, focusing on one database is a potential limitation of this study. Future reviews could search multiple databases to ensure the comprehensiveness of sources included.

### Study characteristics

Of the 33 articles, 28 were published after 2018, reflecting an increased interest and attention in recent years (Supplementary Table 4). The articles were published in 25 journals and cover various research areas.

Most of the studies (*n* = 19) used qualitative methods for analysis, including reviews, focus groups or interviews. Articles embedding the protein transition into a theoretical or conceptual framework, such as the multilevel perspective framework, were categorized under qualitative methodologies. Of the studies, six used only quantitative methods, such as life cycle analysis or flow analysis, and eight used qualitative and quantitative methods, combining survey data and interviews or focus groups. Based on our article selection, qualitative methods are more commonly used than quantitative methods.

### Reporting summary

Further information on research design is available in the Nature Portfolio Reporting Summary linked to this article.

### Supplementary information

Supplementary Information Supplementary Tables 1–7, Fig. 1, coding instructions and PRISMA 2020 checklist.

Reporting Summary

## Data Availability

Data used in this study, including a list of papers reviewed, can be found in Supplementary Information.
