# Supplementary material for: A systematic review of the definitions, narratives and paths forwards for a protein transition in high-income countries
Source: Nat Food. 2024 Jan 3;5(1):28–36. doi: 10.1038/s43016-023-00906-7 (PMC10810756; doi:10.1038/s43016-023-00906-7)
Supplement: Supplementary file 1 — Supplementary Tables 1–7, Fig. 1, coding instructions and PRISMA 2020 checklist. [file 43016_2023_906_MOESM1_ESM.pdf]

nature food

Article https://doi.org/10.1038/s43016-023-00906-7

A systematic review of the definitions, narratives and paths forwards for a protein transition in high-income countries

In the format provided by the authors and unedited

Supplementary material

Supplementary material 1

Table S1: Definitions and interpretations of the ‘protein transition’ or ‘protein shift’ 2

Table S2: Research focus category, research questions and proposed solutions 5

Table S3: References in the introduction. The darker the green, the more cited the reference is. 14

Coding instructions 16

Figure S1: Coding themes: The figure illustrates the nodes associated with various coding themes, with each primary node corresponding to a table in the supplementary data (Tables S1-S7). 19

Table S4: Bibliometrics of research on the protein transition: journals, authors *(*first author)*, affiliations, countries, year of publication and list of reviewed articles 19

Table S5: Geographical scope, focus and methodology used. (*EU is defined by the member states included at different periods.) 20

Table S6: Problem framing (Why is the current situation problematic, and why do we need a transition?) 21

Table S7: Sustainability themes 25

PRISMA 2020 CHECKLIST 26

Table S1: Definitions and interpretations of the 'protein transition' or 'protein shift'

Author, year	Country of first author	Focus	Definition	Definition focus	Reference in the definition
Aiking and de Boer (2020)	The Netherlands	Global issues	"The "protein transition" is about a dietary shift away from meat- and dairy-rich food consumption patterns towards eating more plant-based proteins"	Consumption	
Dagevos (2021)	The Netherlands	Consumers	* "A reverse and more reluctant dietary shift ("a second nutrition transition" – Vranken, Avermaete, Petalios, & Mathijs, 2014) arises in high-income countries away from animal protein products towards plant-based foods."<br>* "The protein transition is about respecting environmental limits rather than outpacing our planet's resources, and is also about following national dietary guidelines rather than ignoring these health recommendations"	Consumption<br>Environmental limits	
Dagevos and Verbeke (2022)	The Netherlands	Consumption and consumers	"protein transition (i.e. shifting diets away from high in animal proteins towards higher in plant proteins)"	Consumption	
de Bakker and Dagevos (2012)	The Netherlands	Consumers	"a protein transition, i.e., a change of meat-centered diets to more plant-based diets (De Bakker and Dagevos 2010: 48–53)."	Consumption	De Bakker and Dagevos (2010)
de Boer and Aiking (2018)	The Netherlands	Consumption and consumers	* "Healthy, pro-environmental protein consumption requires a transition to a diet with more plant protein and considerably less animal protein."<br>* "a 'reversed' diet transition- a shift to a diet with more plant protein and considerably less animal protein."	Consumption	
de Boer and Aiking (2019)	The Netherlands	Consumption and consumers	"The transition aims to reduce total protein intake as well as the dietary ratio of animal over plant protein (from 60:40 via 50:50 to 40:60), which will require changes in consumer food choice processes at the levels of diets, dishes and dish ingredients."	Consumption	
de Boer and Aiking (2011)	The Netherlands	Global issues and consumption	* "This may enable a novel protein transition, featuring a greater share of plant-based protein."<br>* "This shed new light on the nutritional transition in the past century that made animals the chief source of protein in Western countries. In view of the impacts, another transition seems to be required, this time to decrease the consumption of animal-based protein and to replace it by plant-based protein."	Consumption	
Duluins et al. (2022)	Belgium	Producers	* "The protein transition, defined as the rebalancing between animal and alternative proteins in diets, is presented as a solution to mitigate the harmful effects of cattle production on the environment, but also as an opportunity to induce healthier diets."<br>* "The protein transition, defined as a rebalancing of protein consumption between animal and alternative proteins, is increasingly presented as a solution to mitigate the harmful effects of livestock production on the environment (Machovina et al., 2015; Prag and Henriksen, 2020) and on animal welfare (de Boer and Aiking, 2011), but also as an opportunity to restore healthier diets (Friel et al., 2009; de Boer and Aiking, 2017)."	Consumption	Machinova et al. (2015)<br>Prag and Henriksen (2020)<br>de Boer and Aiking (2011)<br>Friel et al. (2009)<br>de Boer and Aiking (2017)

Hartmann and Siegrist (2017)	Switzerland	Consumers	"A transition from animal-based to plant-based proteins would be beneficial for biodiversity, land use, water use, climate, human health and animal welfare (Aiking, 2011; Leip et al., 2015). A reduction in the consumption of meat and dairy products is, therefore, crucial for more sustainable food production (Aiking, 2014; Lamb et al., 2016)."	Consumption Production	
Harwatt (2019)	USA	Production	"Animal to plant-sourced protein shifts offer substantial potential for GHG emission reductions." "This outlook article outlines why animal to plant-sourced protein shifts should be taken up by the Conference of the Parties (COP), and how they could feature as part of countries' mitigation commitments under their updated Nationally Determined Contributions (NDCs) to be adopted from 2020 onwards."	Unclear	
Heerschop et al. (2022)	The Netherlands	Consumption and consumers	"a need for replacement of animal-based foods by plant-based foods," "Overall, these modeled diets show a replacement of animal-based foods by plant-based foods, also called "the protein transition" (6)."	Consumption	Aiking and de Boer (2020)
Hundscheid et al. (2022)	Austria	Transition dynamics	"Despite a small number of countries, which started to set measures supporting a sustainable protein transition (change from a diet with a high proportion of animal proteins to a higher proportion of plant proteins), policy interventions are largely missing in European countries."	Consumption	
Koole (2022)	The Netherlands	Producers	"Several studies have been done into these trends from a transition perspective, in which the veganism trend has been called a 'protein transition', as it may lead to a shift in practices of production and consumption towards less animal and more plant protein (Mylan et al., 2019; Ploll et al., 2020; Tziva et al., 2020)."	Consumption Production	Mylan et al. (2019) Ploll et al. (2020) Tziva et al. (2020)
Leroy et al. (2022)	Belgium	Nutrition	"A striking example of such a counterproductive approach is the excessive projection of contemporary dietary challenges on the notion of protein transition. The latter implies that the human population should shift to diets that restrict 'animal protein' (described usually with connotations of environmental- and health-related harm) and fill in the deficit with 'plant protein', often framed as 'plant-based alternatives' (Willett et al. 2019)."	Consumption	Willett et al. (2019)
Manners et al. (2020)	Rwanda	Transition dynamics	"Meeting the desired objective was considered crucial to enhance protein transition from animal-based to plant-based protein and thus, promote a more sustainable plant-rich diet in the EU."	Consumption	
Onwezen (2022)	The Netherlands	Consumption	"Protein transition, i.e. the transition from high levels of traditional meat consumption towards consuming less meat or more plant-based or alternative animal-based proteins, is highly dependent on consumer behaviour."	Consumption	
Paloviita (2021)	Finland	Consumption and consumers	"Protein transition in diets constitutes eating considerably less animal protein and more plantbased and alternative sources of protein (Ripple et al., 2017; de Boer and Aiking, 2018)."	Consumption	Ripple et al. (2017) de Boer and Aiking (2018)

Tziva et al. (2021)	The Netherlands	Transition dynamics	"Taking these issues into consideration, scientists have increasingly recognized the reduction of animal products consumption and the diffusion of plant-based products, as potential mitigation options (Aiking and de Boer, 2018; Hallstrom et al., 2015; Herrero et al., 2016)."	Consumption	Aiking and de Boer (2018)<br>Hallstrom et al. (2015)<br>Herrero et al. (2016)
Van Den Burg et al. (2021)	The Netherlands	Alternative proteins	"The "protein transition" is about a dietary shift away from meat and dairy-rich food consumption patterns towards eating more plant-based proteins."	Consumption	
van der Weele et al. (2019)	The Netherlands	Alternative proteins	"While a "protein transition" towards meat alternatives would be technologically and organisationally plausible (Hoogland, te Riele, & Rotmans, 2008), barriers emerge from, e.g., the high social status of meat, established economic interests in the meat chain, insufficient technological know-how on novel (plant) protein foods and the optimised use of all by-products in the meat chain (Aiking, 2011)."	Consumption	Hoogland et al. (2008)<br>Aiking (2011)

Table S2: Research focus category, research questions and proposed solutions

Authors	Keywords	Research focus category	Research question(s) or aim(s) of the paper	Proposed solution(s) - everything but consumption-based approaches	Proposed solution(s) - consumption-based approaches	Narrative
aan den Toorn et al. (2020)	Climate change; Industrial ecology; Livestock; Meat and dairy; Socioeconomic metabolism; Supply and use tables	Production GHG	"We aim to address this knowledge gap by quantifying GHG emissions and the economic flows of meat and dairy supply chains."		Animal-based products replacement or substitution	NA
Aiking H. (2011)	NA	Global issues	"This paper sketches the overall playing field of interlinked sustainability issues, the temporal aspects (Aiking & De Boer, 2004), and the pivotal role of nitrogen and protein."	Research and development towards novel protein foods	Information strategies (Consumer education) Coercion and incentivisation (e.g. tax regulations or price promotions) Food waste reduction Animal-based products replacement or substitution Alternative proteins for food	Consumer
Aiking H. (2014)	NA	Global issues	"Therefore, the current article argues that food security, food sustainability, and nutrition are increasingly intertwined and should be addressed in an integrated way, by identifying trade-offs and setting priorities."		Coercion and incentivisation (e.g. tax regulations or price promotions) Animal-based products replacement or substitution Reducing protein consumption (particularly that of animal protein)	Consumer

Aiking H., de Boer J.	NA	Global issues	"This review starts out by identifying ecological, economic and social aspects of sustainable food consumption. In order to address the inefficiencies inherent to current dietary patterns, therefore, a ranked list of more sustainable options is proposed, based on their order of magnitude. Addressing consumers, industry, and governmental stakeholders plus cultural aspects, challenges and options are sketched."		Information strategies (information to increase knowledge or understanding)<br>Food waste reduction<br>Animal-based products replacement or substitution<br>Reducing protein consumption (particularly that of animal protein)<br>Flexitarism	Consumer
Bryant and van der Weele (2021)	Agriculture; Farmers; Farming; Meat; Meat alternatives; Protein transition	Farmers moral ambivalence	"How do farmers respond to the changing atmosphere? Our aim is to elucidate the moral ambivalence farmers and meat production workers feel about their work, and begin a discussion about how we should understand the role of animal farmers in the societal shift away from meat."		Alternative proteins for food	Techno-centred
Dagevos (2021)	Consumer culture; Dietary change; Meat reduction; Protein transition; Sustainable diet	Consumer behaviour	"This study collected recent consumer research on meat eaters and meat reducers conducted in various affluent countries to explore the state of play in the field of flexitarianism."		Animal-based products replacement or substitution<br>Reducing protein consumption (particularly that of animal protein)<br>Flexitarianism	Consumer
Dagevos and Verbeke (2022)	Data; Flexitarianism; Meat consumption; Protein transition; Sustainability	Consumer behaviour	"narrative descriptive review of (reductions in) meat consumption in the Netherlands and Belgium with a focus on trends during the period 2010–2020"		Cultural and habitual shift away from meat centred diets<br>Reducing protein consumption (particularly that of animal protein)<br>Flexitarianism	Consumer

de Bakker and Dagevos (2012)	Consumer society; Ethical consumer; Flexitarianism; Food citizen; Meat; Sustainable consumption; Transition	Consumer behaviour	Yet, the question is whether modern consumers can be considered as reliable allies to achieve this shift in meat consumption pattern. Is there not a yawning gap between our responsible intentions as citizens and our hedonic desires as consumers?		Animal-based products replacement or substitution<br>Smaller portions of meat<br>Meatless days<br>'Less but better meat'<br>Flexitarianism<br>Cultural and habitual shift away from meat centered diets<br>Reducing protein consumption (particularly that of animal protein)	Consumer
de Boer and Aiking (2018)	Animal protein; Plant protein; Health; Environment; Consumers	Consumer behaviour	"The aim is to assess how responses to the options might be shaped by 1) cultural, culinary and economic spatial gradients (including GDP per capita) at regional level and 2) differences in environmental friendly behavior and gender at individual level."	Adapted dietary guidelines<br>Role of retailers	Animal-based products replacement or substitution<br>Reducing protein consumption (particularly that of animal protein)	Consumer
de Boer and Aiking (2019)	Protein; Consumer transition; Health; Sustainability; Dishes	Consumption	"However, rather less information is available about how such changes can be operationalized and implemented at the levels of dishes and dish ingredients. Considering that all these levels are key to a diet shift, the present paper describes the background and the potential use in strategy development of the proposed DDDI (diets, dishes, dish ingredients) framework to support a protein transition."		Information strategies (information to increase knowledge or understanding)<br>Food waste reduction<br>Animal-based products replacement or substitution<br>Meatless days<br>Cultural and habitual shift away from meat centered diets<br>Reducing protein consumption (particularly that of animal protein)<br>Change ratio of animal plant protein<br>Alternative proteins for food	Consumer

de Boer and Aiking (2011)	Plant-based proteins; Meat; Food security; Consumer preferences; Sustainability	Global issues Consumption	"Using a macro perspective, it presents a review of the literature on current and future impacts of the nutritional transition that has made animals the chief source of protein in many countries. Using a micro perspective, survey data on consumers reveal that their frames and habits are strongly adapted to the current meat system." "The aim of the paper is to demonstrate the importance of protein production for the global environment and to give insight into the way consumers frame the protein part of their meal."		Nudging Animal-based products replacement or substitution Cultural and habitual shift away from meat centered diets Reducing protein consumption (particularly that of animal protein) Alternative proteins for food	Consumer
Derler et al. (2021)	By-products; Circular economy; Circular food system; Insect farming; Tenebrio molitor	Alternative proteins	"Following that, we address two research questions in this article. First, which agricultural and industrial by-products have been fed to T. molitor and described in the literature? Second, what are promising fields of application of T. molitor in circular food systems? Thus, this article provides an overview and discusses current and future applications of T. molitor as a biomass converter in a CE."	Alternative proteins for feed Technology and infrastructure Legal framework	Alternative proteins for food	Techno-centred
Detzel et al. (2021)	Life cycle assessment; Plant-based meat substitutes; Plant-based milk substitutes; Protein transition; Protein-rich food; Sustainable food	Alternative proteins	"The Protein2Food prototypes are highly processed foods. One of the guiding questions in the project was whether these innovative plant-based foods are really more environmentally sustainable than the traditional animal-based counterparts. This question was addressed by comparing the environmental footprints of the plant based protein-rich prototypes against animal-based references."		Alternative proteins for food	Techno-centred
Duluins et al. (2022)	Economic performance; Feed self-sufficiency; Grazing practices; Intensification;	Farmers (economic perspective)	"What are the economic implications of a protein transition on livestock farmers?"	Change in production systems (towards more extensive production systems) Change in the		Socio-technological

	Livestock production; Protein transition			choice of breeds<br>Change in feeding strategies		
Hartmann and Siegrist (2017)	Consumer; Environment; Insects; Meat; Meat substitutes; Sustainability	Consumer behaviour	"1) Are consumers aware that meat consumption has a large environmental impact? 2) Are consumers willing to reduce meat consumption or substitute meat with an alternative? 3) Are consumers willing to accept meat substitutes and alternative proteins, such as insects or cultured meat?"	Technology and infrastructure (but insufficient alone)	Nudging<br>Information strategies (information to increase knowledge or understanding)<br>Animal-based products replacement or substitution<br>Reducing protein consumption (particularly that of animal protein)<br>Alternative proteins for food	Consumer
Harwatt (2019)	Animal agriculture; Animal to plant-protein shifts; Climate change mitigation; Climate policy; COP 24; Paris agreement	Production GHG	"Overarching approach to reducing GHG emissions"	Reduce livestock production<br>Favour monogastric over ruminant<br>Change in production systems (towards more agro-ecological conditions)<br>Technology and infrastructure (but insufficient alone)	Animal-based products replacement or substitution	Techno-centred
Heerschop et al. (2022)	Acceptability, meat consumption, diet shift, two-part model, diet transition	Consumer behavior	"To get insight into acceptable substitutions for meat in the diet, this study analyzed dietary patterns to describe what food groups are consumed instead of meat in subgroups of the Dutch population, using data from the Dutch National Food Consumption Survey 2012–2016 (DNFCS)"		Animal-based products replacement or substitution<br>Substitution strategies	Consumer
Hundscheid et al. (2022)	Institutional theory; Media analysis; Multi-level perspective; Protein transition; Sustainable protein consumption	Socio-technical transition	"(1) How did the depiction of meat consumption and alternative protein sources in Austria's print media change between 2000 and 2019? (2) What is the current state of the protein transition in Austria with regard to the four MLP transition phases? (3) Which institutional changes can be detected	Public policy measures<br>New norms, rules, standards and their institutionalization<br>Legal framework<br>Adapted dietary guidelines	Information strategies (information to increase knowledge or understanding)<br>Animal-based products replacement or substitution<br>Meatless days<br>'Less but better	Socio-technological

			working in support or against the protein transition?”		meat' Alternative proteins for food	
Koole (2022)	NA	Socio-technical transition	“Therefore, the main question explored in this article is how trust was actively constructed in the context of the controversy that arose in the interactive learning process within this Swedish ‘reflexive arrangement’, i.e.: a temporary network that focuses on mobilizing actors to establish structural change (see Hendriks and Grin, 2007; Meadowcroft, 2009, Marsden, 2013; Loeber and Vermeulen, 2016).”			Socio-technological
Leroy et al. (2022)	Dairy; Eggs; Livestock; Meat; Plant-based; Poultry; Vegan; Vegetarian	Socio-technical transition	“Motivated by dangers of nutritional mis- and disinformation spreading, particularly given the rapid power of transmission via social media platforms, the present article explores unintended pitfalls of nutritionism approach related to the qualifier ‘animal protein’, hence pre-empting unhelpful conclusions and policies such a school of thought may result in.”	Public policy measures		NA
Lonkila and Kaljonen (2021)	Alternative protein; Literature review; Meat alternative; Milk alternative; Protein transition; Sustainable food system	Alternative proteins	“We review 123 social scientific journal articles on cell-based and plant-based meat and milk alternatives to understand how the positioning of alternatives as both same and different in relation to animal-based products influences their role within the protein transition”	Technological innovation	Reducing protein consumption (particularly that of animal protein) Alternative proteins for food	Techno-centred
Manners et al. (2020)	Backcasting; Future; Meat substitution; Pathways; Plant proteins; Stakeholders	Socio-technical transition	“This analysis looks to: (i) understand the barriers and opportunities that may inhibit or enable dietary and production changes in the future; (ii) map policy actions and milestones needed to achieve a sustainable future; iii) develop stakeholder developed strategies for	New varieties of protein rich crops Research and development on plant proteins/proteins rich crops Public policy measures Supply and value chains	Information strategies (information to increase knowledge or understanding)	Socio-technological

			moving towards this sustainable future.”	Role of retailers<br>Production and consumption changes are needed		
Mylan (2018)	Sustainable consumption and production; Meat; Behaviour change; Food; De-animalisation; Plant-based diet; Flexitarian	Consumer behaviour	In the context of this debate on meat consumption, the paper aims to enrich the understanding of the challenges and strategies used by people who attempt to enact meat-reduced diets.		Planning meals<br>Animal-based products replacement or substitution<br>Reducing protein consumption (particularly that of animal protein)	Consumer
Onwezen (2022)	Acceptance; Behaviour change; Interventions; Meat alternatives; Novel proteins	Consumer behaviour	“The main aim of the current position paper is to demonstrate how research in behavioural sciences can be used to develop interventions towards meat reduction by using four systematic steps.”	Public policy measures	Training and developing cooking skills<br>Persuasion strategies (e.g. highlighting benefits)<br>Nudging<br>Modelling (e.g. inspiring individuals’ consumers)<br>Information strategies (information to increase knowledge or understanding)<br>Coercion and incentivisation (e.g. tax regulations or price promotions)<br>Animal-based products replacement or substitution<br>Smaller portions of meat<br>Reducing protein consumption (particularly that of animal protein)<br>Vegetarian or vegan strategies	Consumer

Paloviita (2021)	Domain; Food system; Governance; Niche; Protein transition; Regime; Scale; Sustainability	Socio-technical transition	“to understand the interdependencies of domains and scales of protein transition towards diets based on plants and alternative sources of proteins.”	Multi-level and multi-domain solutions (including food quality, health, socio-economic and socio-cultural domains)<br>Public policy measures<br>Supply and value chains<br>Production and consumption changes are needed		Socio-technological
Prag and Henriksen (2020)	Sustainable food systems; Agriculture; Livestock; Greenhouse gas emissions; Climate change; Meat; Plant-based; Diet; Scenarios; Policy	Socio-technical transition	“The study develops transition scenarios for a sustainable conversion from animal-based to plant-based food production, associated with a projected increasing global implementation of the PHD.”	Reduce livestock production<br>Agriculture system transition (from production to consumption)<br>Conversion of livestock production systems to plant production systems<br>Alternative proteins for feed<br>Public policy interventions<br>Legal framework<br>Economic incentives	Persuasion strategies (e.g. highlighting benefits)<br>Modelling (e.g. inspiring individuals’ consumers)<br>Information strategies<br>Consumption reduction<br>Coercion and incentivisation (e.g. tax regulations or price promotions)<br>Alternative proteins for food	Socio-technological
Spiller et al. (2020)	Novel protein; Nutrient recovery; Protein transition; Purple phototrophic bacteria; Resource recovery; Single-cell protein	Alternative proteins	“Potential of microbial protein (MP) for feed.”	Alternative proteins for feed		Techno-centred
Tuhumury (2021)	NA	Alternative proteins	“Potential of insects for food and feed.”	Alternative proteins for feed<br>Research and development	Alternative proteins for food	Techno-centred
Tziva M., Negro S.O., Kalfagianni A., Hekkert M.P.	Alliances; Protein transition; Regulatory intermediaries; Sustainability transitions; System building;	Socio-technical transition	“1) How do organizational motives, organizational resources, and relationships influence the formation of alliances? 2) How can alliances contribute to the development of system building strategies and the			Socio-technological

	Technological innovation system		creation of system-level resources?”			
Tziva M., Negro S.O., Kalfagianni A., Hekkert M.P.	Food; Norms; Plant-based proteins; Sustainability transtition; Technological innovation system; Users	Socio-technical transition	“The key question is whether the same mechanisms derived from previous transitions literature also hold for transitions in the food processing industry. In this paper, we study the emergence of the plant-based meat substitutes industry in the Netherlands in order to explore how key innovation processes develop in the food processing sector.”	Research and development<br>Technological innovation	Change ratio of animal plant protein<br>Alternative proteins for food	Socio-technological
Van Den Burg S.W.K., Dagevos H., Helmes R.J.K.	Aquaculture; Circular food systems; Consumers; LCA; seaweed	Alternative proteins	“A question left unanswered is how the European produced seaweeds would fit into the existing global value chains and compete on the world market.”	Alternative proteins for feed	Alternative proteins for food	Techno-centred
van der Weele C., Feindt P., Jan van der Goot A., van Mierlo B., van Boekel M.	Algae; Cultured meat; Innovation; Insects; Plant-based meat alternatives; Protein transition; Pulses; Sustainability	Alternative proteins	“What are the pre-conditions and implications of the various alternatives to meat, what level and kinds of social-institutional change do they imply, and what sustainability gains are they likely to achieve?”	Integrated approaches	Alternative proteins for food	Techno-centred
Weindl et al. (2021)	Dietary requirements; Environmental impacts; Food system; Health; Protein; Sustainable diets	Global issues	No research question. “The aim of the paper is to build on consensus statements on the protein transition shared by participants of a workshop.”	Sustainable aquaculture intensification<br>New varieties of protein rich crops<br>Favour monogastric over ruminants<br>Alternative proteins for feed and food<br>Public policy measures<br>Production and consumption changes are needed<br>Integrated approaches	Change ratio of animal plant protein<br>Alternative proteins for food	Techno-centred

Table S3: References in the introduction. The darker the green, the more cited the reference is.

Most cited references	Number of occurrences	Number of articles mentioning the article	Use of the reference
Aiking & de Boer (2018)	9	9	- Environmental impacts of animal proteins/animal-based production/ livestock (industrial/intensive) farming (3)<br>- Unsustainability of protein production and consumption<br>- Framework used in the article<br>- Consumer directed strategies<br>- Reducing animal products consumption as a mitigation option
Aiking (2011)	7	5	- Environmental impacts of animal proteins/animal-based production/ livestock (industrial/intensive) farming (2)<br>- Unclear<br>- Global food security<br>- Reducing animal products consumption as a mitigation option<br>- Consumer directed strategies<br>- Insufficient technological knowledge
Aiking (2014)	6	5	- Environmental impacts of animal proteins/animal-based production/ livestock (industrial/intensive) farming (3)<br>- Environmental and health impacts of the current ratio between<br>- animal and plant protein<br>- Reducing animal product consumption as a mitigation option of (…)<br>- Change in nutrition behaviour
de Boer and Aiking (2018)	4	3	- Demand for more sustainable and healthier livestock production (2)<br>- Definition of the protein transition<br>- Health and environmental impacts of animal protein
de Boer and Aiking (2019)	4	4	- High processed and red meat intake<br>- Environmental impacts linked to industrial meat production<br>- Framework<br>- Reducing animal protein consumption as a mitigation option of (…)
Gerber et al. (2013)	6	6	- Environmental impacts of animal proteins/animal-based production/ livestock (industrial/intensive) farming (5)
Godfray et al. (2010)	2	2	- Food security (2)<br>- International trade of feed
Godfray et al. (2018)	3	3	- Reducing animal protein consumption as a mitigation option of (…) (2)<br>- Need to rebalance protein intake between animal and plant sources
Grigg (1995)	7	5	- Animals as the chief source of protein in western diets/ nutritional transition (3)<br>- Proteins in the context of culture, habits and environment (2)<br>- Increasing demand for animal proteins<br>- Influence of gradients on pro-environmental protein options
Machinova et al. (2015)	3	3	- Environmental impacts linked to livestock production (2)<br>- Biodiversity

Poore & Nemecek, (2018)	5	6	- Environmental impacts of animal proteins/animal-based production/ livestock (industrial/intensive) farming (3)<br>- Reducing animal protein consumption as a mitigation option of (…)<br>- Animal protein consumption
Rockström et al. (2009)	3	3	- Planetary boundaries (3)
Springmann et al. (2018)	10	5	- Need to rebalance protein intake between animal and plant sources (3)<br>- Reducing animal protein consumption as a mitigation option of (…) (4)<br>- Environmental and health impacts of current consumption (2)<br>- Reducing production and consumption to meet Paris Agreements
Steffen et al. (2015)	3	3	- Planetary boundaries (3)
Steinfield et al. (2006)	12	9	- Environmental impacts of animal proteins/animal-based production/ livestock (industrial/intensive) farming (9)<br>- Growing population and food security (2)<br>- Reducing animal production and consumption as a mitigation option of (…)
Westhoek et al. (2014)	6	5	- Environmental and health impacts of current consumption<br>- Environmental impacts of animal proteins/animal-based production/ livestock (industrial/intensive) farming<br>- Reducing animal protein consumption as a mitigation option of (…) (3)<br>- Health risks related to excessive animal protein intake
Willett et al. (2019)	16	11	- Environmental and health recommendations (9)<br>- Planetary boundaries (2)<br>- Need to rebalance protein intake between animal and plant sources<br>- Reducing animal protein consumption as a mitigation option of (…)<br>- Health risks related to excessive animal protein intake<br>- Environmental impact of animal production and consumption

Coding instructions

1. **Initial Code Set Based on Research Questions:**

During the initial coding phase, we developed a first set of codes based on the research questions and previous knowledge of the literature. The first-order codes included:

- Citations in the introduction: each reference in the introduction should be coded as a new node.
- Definitions and interpretations: all definitions and interpretations of the protein transition should be encoded in this node.
- Methodology: a distinction is made between qualitative and quantitative methodologies. See below for more information regarding the distinction between methodologies*
- Problem framing: This node encompasses all aspects that provide a rationale for the necessity of the protein transition. It includes explanations, reasons, and arguments highlighting why the transition is needed.
- Proposed solutions: Within this node, we capture all elements describing the recommended approaches and actions for the protein transition. It encompasses strategies, interventions, and proposals on effectively implementing the transition, including potential pathways and interventions to achieve the desired objectives.
- Research question or aim: research questions and objectives are coded within this node.
- Geographical scope: the geographical scope of the study encoded in this node.
- Sustainability themes: we used Table S3 from 'Less but Better Meat' (Resare Sahlin et al., 2020) to establish the initial set of sustainability issues.
- Research focus: the main line of research is encoded in this node.**

* Further instructions for coding qualitative vs quantitative methodologies:

For our systematic review, we classified studies as either qualitative or quantitative based on the following criteria:

Quantitative methods included:

- Statistical analysis: Studies that employed statistical techniques to analyse and interpret numerical data related to the protein transition.
- LCA (Life Cycle Assessment): Studies that used LCA methodology to assess the environmental impacts of different protein sources.
- Quantitative survey data: Studies that collected data through structured surveys with quantifiable responses related to the protein transition.
- Flow analysis: Studies that utilised flow analysis methods to understand material and energy flows in the context of the protein transition.
- Econometric model: Studies that employed econometric modelling to analyse economic aspects of the protein transition.

Qualitative methods included:

- Reviews: Studies that provided a comprehensive synthesis and analysis of existing literature on the topic without using quantitative data analysis.
- Event-history timeline: Studies that used a chronological representation of events to understand the historical development of the protein transition.

- Interviews and focus groups: Studies that collected data through interviews or focus group discussions to explore participants' perceptions and experiences related to the protein transition.
- Participatory back-casting: Studies that engaged stakeholders in envisioning and planning for future scenarios related to the protein transition.
- 'Conceptual frameworks': Studies that presented theoretical models or frameworks, such as the multi-level framework, DDPI framework (Diet, dishes, and dish ingredients), heuristic framework on interactions of trust and learning, and matrix framework to understand the interdependencies of domains and scales of protein transition towards diets based on plants and alternative sources of proteins.

** Further instructions for coding the research focus:

Coding instructions for topics covered:

- Consumption and consumers: Consumption and Consumers: Articles in this category primarily focused on consumers and their dietary habits, exploring topics related to changing consumption patterns, nutritional considerations, and health perspectives. The central theme revolved around understanding consumer behaviour and its impact on the protein transition.
- Production and producers: Articles in this category centred on specific production practices and their consequences, such as environmental impacts associated with dairy products or the role of livestock breeders. The main emphasis was on exploring the production side of the protein transition.
- Alternative proteins for food and feed: This category included articles that predominantly discussed alternative protein sources for human and animal consumption, focusing on meat and milk alternatives. While these articles also encompassed production and consumption aspects, their central theme was the potential of alternative food and feed sources.
- Global Issues: Articles falling under this category took a comprehensive view of the protein transition, addressing various topics, including environmental impacts, animal welfare issues, future protein demand, and other global aspects related to current protein production and consumption patterns.
- Transition Dynamics: Articles categorised under this theme specifically adopted theoretical frameworks to study protein transition as a complex and dynamic process. For example, they may have applied the multi-level framework of Geels and Schot (2007) to analyse various dimensions of the transition.
- Nutrition: Articles specifically focusing on the nutritional aspects of the protein transition should be classified within this category. These articles delve into the dietary composition, health implications, and nutritional value of different protein sources.

By organising the articles into these distinct categories, we aim to understand better the diverse focus areas covered in the protein transition literature.

2. **Refinement of Coding:**

Both authors tested the initial coding proposal on five randomly chosen articles. Following this round, we refined the coding by making the following adjustments:

- Adaptation of sustainability themes: based on further analysis, we added new themes, such as deforestation and efficiency, and retrieved themes like eating quality.
- Distinction between problem framing and sustainability issues: After discussions, we determined that elements in the introduction could contribute to both problem framing and

sustainability issues codes. Elements appearing after the introduction were coded under sustainability issues.

3. **Thematic Grouping and Further Analysis:**

The coded text was then transferred to a Word document and thematically grouped for further analysis. We created various tables to organise the data and address specific research questions:

- Table S1: Bibliometrics of research on the protein transition: journals, authors *(*first author)*, affiliations, countries, year of publication and list of reviewed articles
- Table S2: Geographical scope, focus and methodology used. (*EU is defined by the member states included at different periods.)
- Table S3: Definitions and interpretations of the 'protein transition' or 'protein shift'
- Table S4: References in the introduction
- Table S5: Problem framing (Why is the current situation problematic, and why do we need a transition?)
- Table S6: Sustainability themes
- Table S7: Research focus category, research questions and proposed solutions

4. **Identifying Main Targets and Narratives:**

We conducted a multi-step process to identify the three main targets of the protein transition. Firstly, we examined the problem framing codes to identify the most commonly occurring challenges. Secondly, we aggregated additional themes when possible. Lastly, we cross-referenced the sustainability issues to ensure comprehensive coverage in explaining the targets. This analysis culminated in the identification of three main challenges that the protein transition aims to address.

For identifying narratives, we examined the proposed solutions and focused on four main aspects: the driver of change, the main objective of the narrative, action pathways, and solutions. Both authors independently looked for emerging narratives during this process.

Figure S1: Coding themes: The figure illustrates the nodes associated with various coding themes, with each primary node corresponding to a table in the supplementary data (Tables S1-S7).

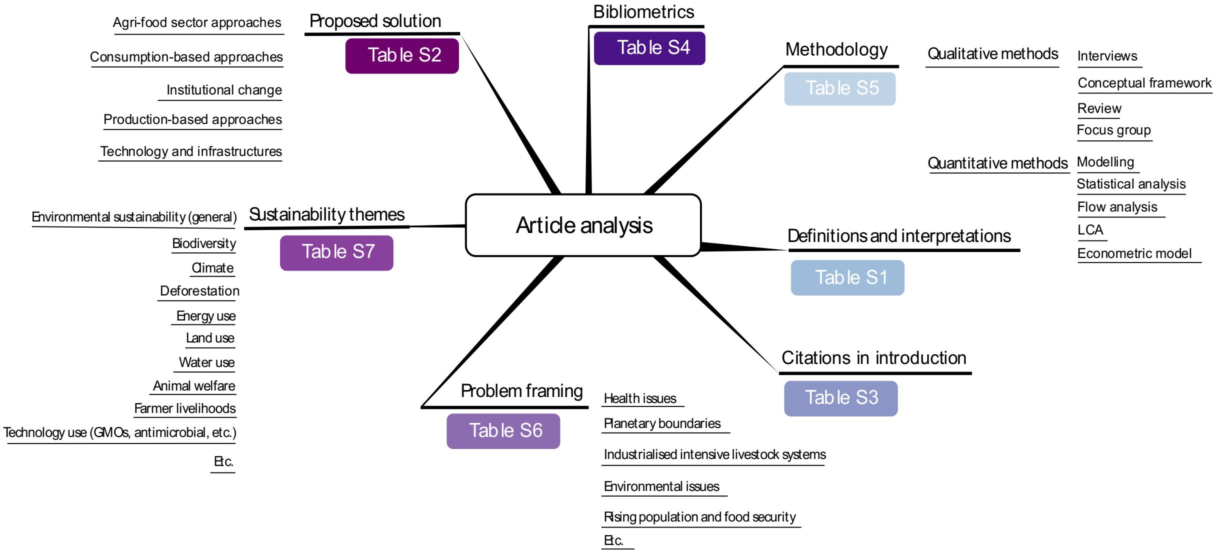

Table S4: Bibliometrics of research on the protein transition: journals, authors *(*first author)*, affiliations, countries, year of publication and list of reviewed articles

Journal	Author	Affiliation	Country	Year of publication
Trends in Food Science and Technology (6)	Aiking H.* (6)	Wageningen University & Research (9)	The Netherlands (22)	2011 (2)
Environmental Innovation and Societal Transitions (2)	de Boer J.* (4)	Leibniz Institute (6)	Germany (15)	2012 (1)
Sustainability (3)	Dagevos H.* (4)	VU University (6)	Belgium (8)	2014 (1)
Appetite (2)	van der Weele C.*(2)	Universidad Politécnica de Madrid (UPM) (5)	Austria (5)	2017 (1)
-	Tziva M.* (2)	University of Antwerp (2)	Spain (5)	2018 (2)
-	Negro S.O. (2)	German Institute of Human Nutrition Potsdam-Rehbruecke (DIfE) (2)	United States (4)	2019 (3)
-	Kalfagianni A. (2)	University of Natural Resources and Life Sciences (2)	UK (3)	2020 (7)
-	Hekkert M.P. (2)	-	-	2021 (9)
-	Blanco-Gutiérrez I. (2)	-	-	2022 (7)
-	Manners R.* (2)	-	-	-

Table S5: Geographical scope, focus and methodology used. (*EU is defined by the member states included at different periods.)

Scope	Methodology	Specifics methodology
The Netherlands (5)	Qualitative (19)	Conceptual and theoretical frameworks (8)
EU (4)*		Review (8)
Western world (3)		Back casting (1)
Austria (2)		Interviews (1)
Belgium (2)		Systematic review (1)
Finland (1)	Quantitative (6)	LCA (2)
France (1)		Flow analysis (1)
Germany (1)		Econometric model (1)
Sweden (1)		Flow analysis and scenario (1)
Denmark (1)		Statistical analysis (1)
UK (1)	Qualitative and quantitative (8)	Statistical analysis and review (3)
World (1)		Survey data and framework (1)
NA (10)		Survey data and interviews (1)
		Survey data and review (1)
		Survey data and review + focus group (1)
		Survey data and focus group (1)

Table S6: Problem framing (Why is the current situation problematic, and why do we need a transition?)

Dimension put forward	Main arguments	# articles	Articles	# occurrence
Animal welfare	- Current livestock production systems are associated with low animal welfare<br>- Moral concerns about the treatment of animals	7	Bryant and van der Weele (2021); Dagevos (2021); Lonkila and Kaljonen (2021); Onwezen (2022); Tuhumury (2021); Tziva et al. (2021); van der Weele et al. (2019)	9
Biodiversity	- Livestock products have disproportionate impacts on biodiversity loss<br>- Livestock production and consumption are responsible for habitat loss, the destruction of terrestrial ecosystems and biological diversity	5	Aiking (2011); de Boer and Aiking (2019), Manners et al. (2021); Tuhumury (2021); van der Weele et al. (2019)	6
Climate	- Livestock production is associated with high GHG emissions (and thus climate change)<br>- Ruminant production has a higher impact than monogastric on climate change	17	aan den Toorn et al. (2020); Aiking (2011); Aiking (2014); de Boer and Aiking (2019); Derler et al. (2021); Godfray et al. (2018); Harwatt (2019); Heerschop et al. (2022); Hundscheid et al. (2022); Lonkila and Kaljonen (2021); Machinova et al. (2015); Manners et al. (2020); Paloviita (2021); Prag and Henriksen (2020); Tuhumury (2021); Tziva et al. (2021); van der Weele et al. (2019)	22
Energy	- Environmental impacts of meat production include the significant use of energy	1	Mylan et al. (2018)	1
Environmental issues (general)	- Environmental pollution<br>- Environmental impacts of livestock production systems and animal-based products consumption<br>- Negative environmental impacts of feed (e.g. soybean)	20	Aiking (2011); Bryant and van der Weele (2021); Dagevos (2021); de Bakker and Dagevos (2012); de Boer and Aiking (2018); de Boer and Aiking (2019); Derler et al. (2021); Detzel et al. (2021); Duluins et al. (2022); Hartmann and	33

			Siegrist (2017); Heerschop et al. (2022); Hundscheid et al. (2022); Leroy et al. (2022); Manners et al. (2020); Onwezen (2022); Paloviita (2021); Prag and Henriksen (2020); Tuhumury (2021); Weindl et al. (2020)	
Health issues	- Animal products overconsumption is associated with adverse health impacts (especially red and processed meat rich in saturated fat)<br>- Shifting away from meat-heavy diets is beneficial for personal health<br>- Including protein in a healthy diet<br>- Current consumption rates exceed health recommendations	18	Bryant and van der Weele (2021); Dagevos (2021); Dagevos and Verbeke (2022); de Boer and Aiking (2018); de Boer and Aiking (2019); Detzel et al. (2021); Duluins et al. (2022); Heerschop et al. (2022); Hundscheid et al. (2022); Leroy et al. (2022); Lonkila and Kaljonen (2021); Manners et al. (2020); Onwezen (2022); Paloviita (2021); Tuhumury (2021); van der Weele et al. (2019); Weindl et al. (2020)	27
Industrialised/intensive livestock systems	Intensive production systems are associated with negative externalities such as:<br>- antibiotic resistance and increasing incidence of emerging diseases;<br>- environmental impacts related to soil, air and water pollution, land use for feed, nitrogen cycle disruption, climate change and biodiversity loss;<br>- unacceptable living conditions<br>Intensive production allows current consumption rates and dietary imbalance	9	Aiking and de Boer (2020); Aiking (2014), Bryant & van der Weele (2021); de Boer and Aiking (2011); de Boer and Aiking (2018); de Boer and Aiking (2019); Duluins et al. (2022); Spiller et al. (2020); Tuhumury (2021)	10
Inefficient protein conversion	- The conversion of feed crops into animal protein is inefficient from a human point of view<br>- Alternatives proteins are more resource efficient	6	de Boer and Aiking (2011); de Boer and Aiking (2018); Derler et al. (2021); Lonkila and Kaljonen (2021); Paloviita (2021); van der Weele et al. (2019)	6

Land use	- More food must be produced on less land<br>- Livestock production requires significant use of land	4	Derler et al. (2021); Mylan (2018); Tuhumury (2021); van der Weele et al. (2019)	4
Planetary boundaries	- (Animal) protein production and consumption are driving the transgression of planetary boundaries<br>- We are operating out of the safe operating space<br>- Shifting away meat-heavy diets is beneficial to stay within planetary boundaries<br>- Future food systems should allow staying within planetary boundaries	10	Aiking and de Boer (2020); Aiking (2011); Dagevos (2021); Dagevos and Verbeke (2022); de Boer and Aiking (2011); de Boer and Aiking (2019); Derler et al. (2021); Detzel et al. (2021); Heerschop et al. (2022); Weindl et al. (2020)	18
Pollution, degradation	- Livestock production is responsible for both resource depletion and pollution	6	Aiking (2011); Manners et al. (2020); Mylan (2018); Tuhumury (2021); Tziva et al. (2021); van der Weele et al. (2019)	6
Protein overconsumption	- Western diets are associated with high animal protein intake, generally exceeding dietary recommendations	5	Aiking (2011); Dagevos and Verbeke (2022); de Boer and Aiking (2019); Hundscheid et al. (2022); van der Weele et al. (2019)	6
Resources scarcity	- Livestock production is responsible for depleting resources that are scarce (including, for example, freshwater or energy)	3	Aiking (2011); Derler et al. (2021); Weindl et al. (2020)	3
Rising population and food security	- Global population is expected to increase - 9 billion in 2050 (and so is the demand for animal proteins)<br>- Food security is at risk<br>- We need to reduce meat production and consumption to ensure global food security	14	Aiking and de Boer (2020); Aiking (2011); Aiking (2014); Dagevos (2021); Dagevos and Verbeke (2022); de Bakker and Dagevos (2012); de Boer and Aiking (2011); Derler et al. (2021); Detzel et al. (2021); Heerschop et al. (2022); Manners et al. (2020); Spiller et al. (2020); Tuhumury (2021); Weindl et al. (2020)	27

Sustainable Development Goals (SDGs)	- Reaching the SDGs requires shifting consumption patterns and production methods, and includes, amongst others the need for a protein transition.	4	Aiking and de Boer (2020); Heerschop et al. (2022); Hundscheid et al. (2022); Manners et al. (2020)	5
Soil	- Livestock production is causing soil loss through erosion	1	Tuhumury (2021)	1
Water depletion and pollution	- Livestock production is associated with high use of water and water pollution	5	Aiking (2011); Aiking (2014); Mylan (2018); Tuhumury (2021); van der Weele (2019)	5

Table S7: Sustainability themes

Most cited themes: animal welfare, biodiversity, environmental sustainability (general), human health, land requirement, nutritional quality and water use.

Sustainability theme	# articles	# references
Air pollution	4	4
Animal welfare and health	18	37
Biodiversity	17	36
Circular food systems	3	8
Climate	28	90
Culture	10	22
Deforestation	5	8
Economy	15	31
Efficiency		
Protein conversion efficiency	10	14
Resource efficiency	4	5
Energy use	11	19
Environmental sustainability (general)	29	115
Ethics	10	20
Farmer livelihoods	5	9
Food safety	2	2
Food security	15	34
Human health	28	117
Industrial livestock production systems	11	17
Land use		
Land requirement	20	40
Feed food competition	5	9
Nutrient cycles	11	18
Nutritional quality	12	38
Pesticides	1	1
Planetary boundaries	6	15
Social	2	6
Technology use (GMOs, antimicrobials, etc.)	7	13
Water use	16	33

PRISMA 2020 CHECKLIST

Section and Topic	Item #	Checklist item	Location where item is reported
**TITLE**			
Title	1	Identify the report as a systematic review.	Line 1-2
**ABSTRACT**			
Abstract	2	See the PRISMA 2020 for Abstracts checklist.	Line 39-52
**INTRODUCTION**			
Rationale	3	Describe the rationale for the review in the context of existing knowledge.	Line 64-73<br>Line 74-77<br>Line 86-88
Objectives	4	Provide an explicit statement of the objective(s) or question(s) the review addresses.	Line 89-93
**METHODS**			
Eligibility criteria	5	Specify the inclusion and exclusion criteria for the review and how studies were grouped for the syntheses.	Line 307-308, Table 4
Information sources	6	Specify all databases, registers, websites, organisations, reference lists and other sources searched or consulted to identify studies. Specify the date when each source was last searched or consulted.	Line 297
Search strategy	7	Present the full search strategies for all databases, registers and websites, including any filters and limits used.	Line 297-305,<br>Line 331-332, Table 4
Selection process	8	Specify the methods used to decide whether a study met the inclusion criteria of the review, including how many reviewers screened each record and each report retrieved, whether they worked independently, and if applicable, details of automation tools used in the process.	Line 300-305,<br>Line 307-308,<br>Line 324-328,<br>Figure 1
Data collection process	9	Specify the methods used to collect data from reports, including how many reviewers collected data from each report, whether they worked independently, any processes for obtaining or confirming data from study investigators, and if applicable, details of automation tools used in the process.	Line 310-322<br>Line 324-328,<br>Supplementary material, Coding instructions
Data items	10a	List and define all outcomes for which data were sought. Specify whether all results that were compatible with each outcome domain in each study were sought (e.g. for all measures, time points, analyses), and if not, the methods used to decide which results to collect.	Line 320-322
	10b	List and define all other variables for which data were sought (e.g. participant and intervention characteristics, funding sources). Describe any assumptions made about any missing or unclear information.	N/A
Study risk of bias assessment	11	Specify the methods used to assess risk of bias in the included studies, including details of the tool(s) used, how many reviewers assessed each study and whether they worked independently, and if applicable, details of automation tools used in the process.	N/A
Effect measures	12	Specify for each outcome the effect measure(s) (e.g. risk ratio, mean difference) used in the synthesis or presentation of results.	N/A
Synthesis methods	13a	Describe the processes used to decide which studies were eligible for each synthesis (e.g. tabulating the study intervention characteristics and comparing against the planned groups for each synthesis (item #5)).	Line 300-305,<br>Line 307-308,<br>Line 324-328,<br>Figure 1
	13b	Describe any methods required to prepare the data for presentation or synthesis, such as handling of missing summary statistics, or data conversions.	Lines 310-315<br>Supplementary data, Coding instructions

Section and Topic	Item #	Checklist item	Location where item is reported
	13c	Describe any methods used to tabulate or visually display results of individual studies and syntheses.	Line 316-322
	13d	Describe any methods used to synthesize results and provide a rationale for the choice(s). If meta-analysis was performed, describe the model(s), method(s) to identify the presence and extent of statistical heterogeneity, and software package(s) used.	Line 320-322, Table S1 to S7
	13e	Describe any methods used to explore possible causes of heterogeneity among study results (e.g. subgroup analysis, meta-regression).	N/A
	13f	Describe any sensitivity analyses conducted to assess robustness of the synthesized results.	N/A
Reporting bias assessment	14	Describe any methods used to assess risk of bias due to missing results in a synthesis (arising from reporting biases).	Line 324-328
Certainty assessment	15	Describe any methods used to assess certainty (or confidence) in the body of evidence for an outcome.	N/A
**RESULTS**			
Study selection	16a	Describe the results of the search and selection process, from the number of records identified in the search to the number of studies included in the review, ideally using a flow diagram.	Figure 1
	16b	Cite studies that might appear to meet the inclusion criteria, but which were excluded, and explain why they were excluded.	N/A
Study characteristics	17	Cite each included study and present its characteristics.	Line 334-341
Risk of bias in studies	18	Present assessments of risk of bias for each included study.	N/A
Results of individual studies	19	For all outcomes, present, for each study: (a) summary statistics for each group (where appropriate) and (b) an effect estimate and its precision (e.g. confidence/credible interval), ideally using structured tables or plots.	N/A
Results of syntheses	20a	For each synthesis, briefly summarise the characteristics and risk of bias among contributing studies.	N/A
	20b	Present results of all statistical syntheses conducted. If meta-analysis was done, present for each the summary estimate and its precision (e.g. confidence/credible interval) and measures of statistical heterogeneity. If comparing groups, describe the direction of the effect.	N/A
	20c	Present results of all investigations of possible causes of heterogeneity among study results.	N/A
	20d	Present results of all sensitivity analyses conducted to assess the robustness of the synthesized results.	N/A
Reporting biases	21	Present assessments of risk of bias due to missing results (arising from reporting biases) for each synthesis assessed.	N/A
Certainty of evidence	22	Present assessments of certainty (or confidence) in the body of evidence for each outcome assessed.	N/A
**DISCUSSION**			
Discussion	23a	Provide a general interpretation of the results in the context of other evidence.	Line 94-100
	23b	Discuss any limitations of the evidence included in the review.	Line 94-100<br>Line 360-363
	23c	Discuss any limitations of the review processes used.	Line 289-295<br>Line 331-332
	23d	Discuss implications of the results for practice, policy, and future research.	Line 50-52,<br>Line 239-245,

Section and Topic	Item #	Checklist item	Location where item is reported
			Line 271-278, Table 2
**OTHER INFORMATION**			
Registration and protocol	24a	Provide registration information for the review, including register name and registration number, or state that the review was not registered.	N/A
	24b	Indicate where the review protocol can be accessed, or state that a protocol was not prepared.	Line 294-295
	24c	Describe and explain any amendments to information provided at registration or in the protocol.	N/A
Support	25	Describe sources of financial or non-financial support for the review, and the role of the funders or sponsors in the review.	Line 349-351
Competing interests	26	Declare any competing interests of review authors.	Line 356-357
Availability of data, code and other materials	27	Report which of the following are publicly available and where they can be found: template data collection forms; data extracted from included studies; data used for all analyses; analytic code; any other materials used in the review.	Line 342-343
